# Testosterone Replacement Therapy in Athletes: Implications for Injury Recovery and Musculoskeletal Performance

**DOI:** 10.7759/cureus.103530

**Published:** 2026-02-13

**Authors:** Luis M Canal de Velasco, José Emiliano González Flores, Alberto Azcona Cervera, Jose Luis Morales Arteaga, María J Olivares Casas

**Affiliations:** 1 Medical Education and Simulation, Panamerican University, Mexico City, MEX; 2 Surgery, Tecnológico de Monterrey Campus Ciudad de Mexico, Mexico City, MEX; 3 Traumatology and Orthopedics, American British Cowdray (ABC) Hospital, Santa Fe Campus, Mexico City, MEX; 4 Cardiology, Panamerican University, Mexico City, MEX

**Keywords:** athletes, muscle hypertrophy, muscle recovery, performance enhancement, sports injury rehabilitation, testosterone replacement therapy

## Abstract

Testosterone is a central regulator of skeletal muscle protein synthesis, connective tissue remodeling, bone homeostasis, erythropoiesis, and systemic metabolic function. Although testosterone replacement therapy (TRT) is an established treatment for male hypogonadism, its role in athletes and physically active populations, particularly in injury recovery and rehabilitation, remains controversial due to limited athlete-specific evidence and ethical and regulatory considerations. Because TRT indications and most outcome data are based on male endocrine reference ranges, the evidence synthesized in this review is predominantly derived from male cohorts. Therapeutic testosterone use in female athletes remains comparatively understudied and cannot be directly generalized given sex-specific physiology, hormonal baselines, and dosing frameworks. We conducted a semi-systematic narrative review of peer-reviewed literature (1990-December 2025) across PubMed/MEDLINE, Embase, Scopus, Web of Science, and the Cochrane Library, complemented by manual reference screening. Eligible publications included clinical studies, observational investigations, translational models, reviews, and consensus statements addressing testosterone or TRT in musculoskeletal recovery, rehabilitation, and sports medicine contexts. Study selection followed a Preferred Reporting Items for Systematic reviews and Meta-Analyses-informed framework, yielding 25 studies for qualitative synthesis. Mechanistic evidence indicates that testosterone promotes skeletal muscle adaptation through androgen receptor signaling and anabolic pathways such as AKT-mTORC1 and insulin-like growth factor-1, while suppressing catabolic regulators including myostatin. Testosterone also enhances satellite cell activation, collagen turnover, bone formation, and tendon remodeling, with additional immunomodulatory effects that support tissue repair. Clinically, supportive data derive largely from hypogonadal, aging, or high-catabolic populations, suggesting that physiological testosterone restoration may mitigate disuse atrophy and improve recovery; however, prospective evidence in injured athletic cohorts remains limited. Ergogenic effects are most consistent for strength and power, whereas endurance outcomes are variable. Current evidence supports testosterone as a permissive modulator of musculoskeletal recovery rather than a universal performance strategy. TRT should be considered a targeted, medically supervised intervention integrated within rehabilitation, with strict regulatory oversight in competitive sport. Future athlete-focused studies are required to define indications, efficacy, and long-term safety.

## Introduction and background

Testosterone is a key anabolic hormone regulating skeletal muscle protein synthesis, bone density, erythropoiesis, and physical performance. Physiological levels peak in early adulthood and decline gradually with age, while conditions such as obesity, metabolic syndrome, and chronic disease can accelerate this decline [1]. In athletes, maintaining optimal testosterone levels is essential not only for overall health but also for sustaining muscle strength, lean mass, and recovery following strenuous activity or injury [2].

Sports-related injuries remain a leading cause of morbidity among athletes. Recovery depends on the coordinated influence of rehabilitation, nutrition, and endocrine function. Testosterone deficiency, whether absolute or relative, has been associated with impaired muscle repair, reduced lean mass, and delayed rehabilitation [3,4].

Testosterone replacement therapy (TRT) has shown efficacy in treating male hypogonadism and improving muscle mass, strength, and quality of life [4]. These effects have prompted interest in its potential role in sports medicine, particularly for enhancing recovery after musculoskeletal injury. Experimental data suggest that testosterone may promote myofiber regeneration, tendon remodeling, and reduce catabolic effects during immobilization or surgical recovery [5].

From a sex-specific applicability perspective, it is important to note that clinical indications for TRT and the majority of outcome data are derived from male hypogonadal populations. While testosterone physiology is relevant in female athletes, therapeutic testosterone use for musculoskeletal recovery in women remains comparatively understudied and must be interpreted within distinct endocrine reference ranges, dosing paradigms, and safety frameworks. Accordingly, extrapolation of TRT-based recovery outcomes from male cohorts to female athletes should be approached with caution.

In clinical practice, “physiological” testosterone refers to concentrations within the expected adult reference range, whereas androgen deficiency implies consistently low levels accompanied by compatible symptoms and/or functional impairment. Importantly, some athletes may develop functional or relative suppression of the hypothalamic-pituitary-gonadal axis despite being young and otherwise healthy. Contributing factors include low energy availability (including relative energy deficiency in sport), high cumulative training load, inadequate recovery, and sustained physiological stress. In these contexts, reduced testosterone may represent an adaptive endocrine response that can intersect with injury recovery capacity and rehabilitation outcomes.

However, the application of TRT in athletes remains controversial due to ethical, regulatory, and safety concerns. Evidence supporting its use in eugonadal or non-elite athletes is limited and inconsistent, and long-term risks such as cardiovascular events or gonadal suppression remain debated [6]. This narrative review aims to critically evaluate the role of TRT in athletes, focusing on its effects on sports injury outcomes and post-injury muscle recovery, and to outline its therapeutic potential, limitations, and ethical implications within sports medicine.

## Review

Methodology

A semi-systematic narrative review approach was adopted to synthesize the available evidence regarding TRT and its role in musculoskeletal recovery and injury rehabilitation in athletes and physically active populations. This approach was intentionally selected due to the substantial heterogeneity across study populations, interventions, outcomes, and study designs, which precluded a fully systematic review or quantitative synthesis.

Literature Search Strategy

A comprehensive and structured literature search was conducted across PubMed/MEDLINE, Embase, Scopus, Web of Science, and the Cochrane Library to identify relevant publications addressing TRT and musculoskeletal recovery. Studies published between January 1990 and December 2025 were considered to capture both foundational experimental research on testosterone physiology and contemporary clinical applications within sports medicine. To enhance completeness and minimize publication bias, reference lists of key articles were manually screened, and citation tracking was performed.

The search strategy combined Medical Subject Headings (MeSH) and free-text terms, including “testosterone replacement therapy,” “TRT,” “athletes,” “sports,” “muscle recovery,” “sports injury,” “rehabilitation,” and “performance enhancement.” Boolean operators (AND/OR) and database-specific filters were applied as appropriate. Only peer-reviewed articles published in English were included.

Study Selection and Eligibility Criteria

Eligible publications included clinical trials, observational studies, experimental and translational models, systematic and narrative reviews, and position or consensus statements that examined the effects of testosterone or TRT on musculoskeletal recovery, injury healing, rehabilitation outcomes, or related physiological mechanisms in athletes or physically active adults. Studies focused exclusively on age-related or primary hypogonadism without relevance to physical performance, injury, or sports medicine contexts were excluded. Non-peer-reviewed sources, including conference abstracts, editorials, and commentaries, were also excluded.

Sex-specific applicability was extracted when reported. However, because TRT clinical indications and most outcomes data are based on male endocrine reference ranges, the included evidence base was predominantly derived from male cohorts. Evidence addressing therapeutic testosterone use in female athletes was limited; therefore, conclusions regarding female applicability were interpreted separately, and extrapolation from male data was approached with caution.

Titles and abstracts were independently screened by two reviewers to assess relevance. Full-text articles meeting the inclusion criteria were subsequently reviewed in detail. Disagreements at any stage were resolved through consensus discussion.

Semi-Systematic Framework and Preferred Reporting Items for Systematic Reviews and Meta-Analyses-Like Reporting

To enhance transparency and reproducibility, elements of systematic review methodology were incorporated, including a structured search strategy, predefined eligibility criteria, and a transparent study selection process. The initial database search identified 312 records, of which 86 duplicates were removed. The remaining 226 records were screened at the title and abstract level, resulting in the exclusion of 179 studies that did not meet the inclusion criteria.

**Figure 1:**
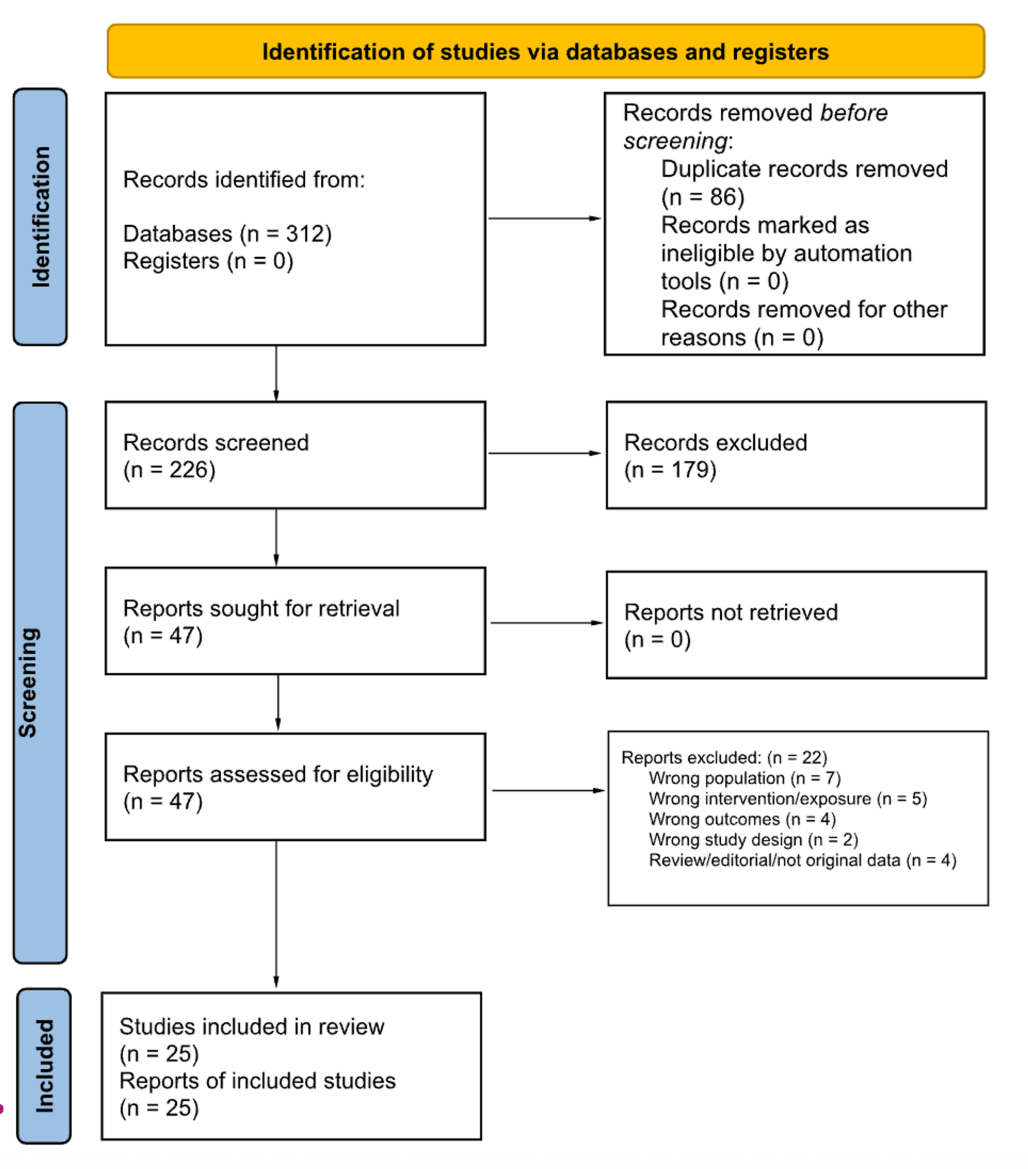
Preferred Reporting Items for Systematic Reviews and Meta-Analyses (PRISMA) 2020 flow diagram illustrating the study selection process for the narrative review. The diagram summarizes records identified through database searches, screening, eligibility assessment, and inclusion in the qualitative synthesis.

Full-text assessment was performed for 47 articles, of which 25 studies met the eligibility criteria and were included in the final qualitative synthesis. The study selection process is summarized using a Preferred Reporting Items for Systematic Reviews and Meta-Analyses (PRISMA)-inspired flow diagram illustrating records identified, screened, assessed for eligibility, and included in the narrative synthesis in Figure 1.

This PRISMA-like figure was employed as a reporting tool to clarify study selection and enhance methodological transparency, rather than as an assertion of a fully systematic review methodology. Given the narrative and integrative nature of this review, no formal quantitative synthesis or meta-analysis was performed.

Quality Appraisal and Data Synthesis

A formal risk-of-bias assessment using standardized tools (e.g., Cochrane Risk of Bias or Newcastle-Ottawa Scale) was not conducted, as such instruments were not uniformly applicable across the diverse study designs included. Instead, studies were qualitatively appraised based on methodological clarity, study design, sample size, population relevance, and level of evidence, in accordance with the Oxford Centre for Evidence-Based Medicine guidelines (2020 update).

Extracted data were organized into predefined thematic domains encompassing molecular and cellular mechanisms of testosterone signaling in skeletal muscle adaptation, including interactions with resistance training and anabolic signaling pathways; effects of testosterone on musculoskeletal integrity, with specific emphasis on tendon remodeling, bone metabolism, and tendon-bone healing following injury or surgical reconstruction; immunomodulatory and anti-inflammatory roles of testosterone in tissue repair and recovery; clinical and observational evidence of TRT in athletes and physically active populations, including implications for injury recovery and rehabilitation; ergogenic and systemic performance effects of testosterone relevant to strength, power, endurance, and metabolic adaptation; and ethical, regulatory, and clinical considerations governing TRT use in athletic settings.

An integrative narrative synthesis was performed to contextualize mechanistic findings alongside clinical and performance-related evidence, while explicitly acknowledging current evidence gaps, methodological heterogeneity, and limitations in athlete-specific data.

Central body

Molecular and Cellular Mechanisms of Testosterone in Skeletal Muscle Adaptation

Testosterone exerts its anabolic effects in skeletal muscle primarily through binding to the androgen receptor, a ligand-activated nuclear transcription factor expressed in both mature myocytes and satellite cells [7-9]. Upon activation, the AR translocates to the nucleus and regulates gene transcription programs involved in myofibrillar protein synthesis, ribosomal biogenesis, and myotube enlargement, thereby directly promoting muscle fiber hypertrophy and structural adaptation [7,10,11]. Beyond its genomic actions, testosterone also modulates non-genomic signaling pathways that contribute to rapid anabolic responses during training and recovery.

At the intracellular signaling level, testosterone enhances activation of the AKT-mTORC1 pathway, a central regulator of muscle protein synthesis and cellular growth. This effect is mediated both directly and indirectly through increased expression and bioavailability of anabolic mediators such as insulin-like growth factor-1 (IGF-1), while concurrently suppressing catabolic signaling pathways, including the ubiquitin-proteasome system and inhibitory regulators such as myostatin [7-9]. Through this coordinated balance between anabolic stimulation and catabolic suppression, testosterone facilitates net protein accretion and supports efficient muscle remodeling during periods of mechanical loading, immobilization, or post-injury recovery.

Human studies provide robust evidence of a dose-response relationship between testosterone exposure and skeletal muscle adaptation. In eugonadal men, graded testosterone administration produces proportional increases in fat-free mass, muscle cross-sectional area, and maximal strength, with more pronounced anabolic effects observed when testosterone supplementation is combined with resistance training [7,8]. These findings highlight testosterone’s permissive role in amplifying training-induced hypertrophy rather than acting as an isolated anabolic stimulus. In hypogonadal and older men, TRT similarly enhances muscle protein synthesis, lean mass, and strength, confirming the critical contribution of adequate androgen signaling to rehabilitation and exercise adaptation [8,9].

At the cellular level, testosterone expands the satellite cell pool and promotes satellite cell activation, proliferation, and differentiation, resulting in increased myonuclear accretion within muscle fibers [8,10]. This process is essential for sustaining larger fiber size and maintaining long-term synthetic capacity during hypertrophy and regeneration. Conversely, experimental suppression of endogenous testosterone has been shown to blunt hypertrophic responses to resistance training, underscoring androgen signaling as a key modulator of skeletal muscle plasticity and adaptive remodeling [11].

**Figure 2:**
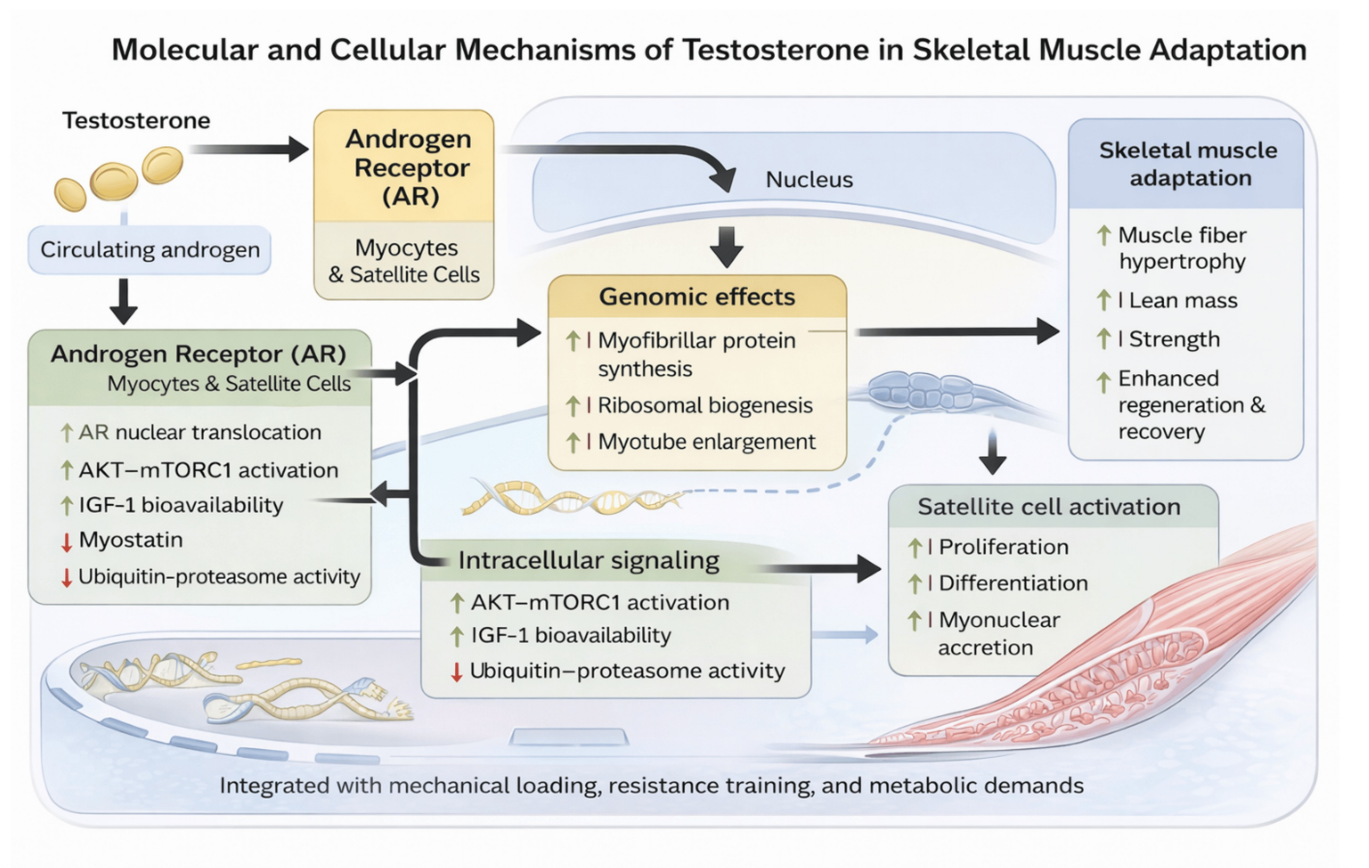
Molecular and cellular mechanisms of testosterone in skeletal muscle adaptation. Schematic overview of the genomic and non-genomic pathways through which testosterone regulates skeletal muscle hypertrophy and remodeling, including androgen receptor activation in myocytes and satellite cells, modulation of AKT–mTORC1 signaling, insulin-like growth factor-1 (IGF-1) bioavailability, suppression of catabolic pathways, and promotion of satellite cell activation and myonuclear accretion. Information was synthesized from references [7-11]. Image credits: The image was created by the authors using BioRender.

Collectively, these molecular and cellular mechanisms position testosterone as a central regulator of skeletal muscle adaptation, integrating hormonal signaling with mechanical loading and metabolic demands (Figure 2). While much of the mechanistic and dose-response evidence derives from controlled studies in non-athletic populations, the pathways illustrated in Figure 2 provide a strong biological rationale for exploring the role of testosterone and TRT in muscle recovery and rehabilitation within athletic contexts.

Testosterone, Resistance Training, and Muscle Hypertrophy

In eugonadal individuals, exogenous testosterone can further augment skeletal muscle hypertrophy beyond baseline physiological adaptations by amplifying the anabolic response to mechanical loading. Controlled trials in men demonstrate a clear dose-response relationship between testosterone exposure and increases in lean body mass, muscle cross-sectional area, and maximal strength, with the most pronounced effects observed when androgen administration is combined with structured resistance training [7,8,10]. These findings indicate that testosterone does not merely act as an independent anabolic stimulus but rather functions as a potent permissive and amplifying factor that enhances training-induced muscle adaptation.

Resistance training provides the primary mechanical signal for muscle hypertrophy through mechanotransduction pathways that activate protein synthesis and satellite cell recruitment. Testosterone synergistically augments these processes by increasing anabolic signaling efficiency, expanding the pool of responsive muscle fibers, and sustaining a positive net protein balance during repeated training bouts. This interaction explains why testosterone supplementation produces disproportionately greater hypertrophic and strength gains when paired with resistance exercise compared to either intervention alone [7-9]. Importantly, this synergy underscores the concept that hormonal milieu critically modulates the magnitude of adaptive responses to identical mechanical stimuli.

Supraphysiologic testosterone administration has been shown to induce significant increases in muscle size and strength even in the absence of exercise, highlighting the intrinsic anabolic potency of androgen signaling [7,8]. However, when supraphysiologic androgen exposure is combined with resistance training, hypertrophic and strength outcomes are markedly amplified, resulting in substantial improvements in one-repetition maximum (1RM), muscle fiber cross-sectional area, and overall neuromuscular performance compared to training alone [9,12]. These observations reinforce the dominant role of testosterone in regulating muscle protein accretion while simultaneously illustrating the additive, and often multiplicative, effects of mechanical loading.

Despite consistent group-level effects, interindividual variability in hypertrophic response to testosterone and resistance training is substantial. Genetic factors, including androgen receptor expression and polymorphisms, differences in baseline hormonal milieu, training status, and metabolic environment, contribute to heterogeneous responsiveness across individuals [10,13,14]. Such variability partially explains why comparable androgen exposure and training regimens yield divergent outcomes in muscle mass and strength, and highlights the importance of individualized considerations when interpreting the effects of testosterone on muscle adaptation.

**Figure 3:**
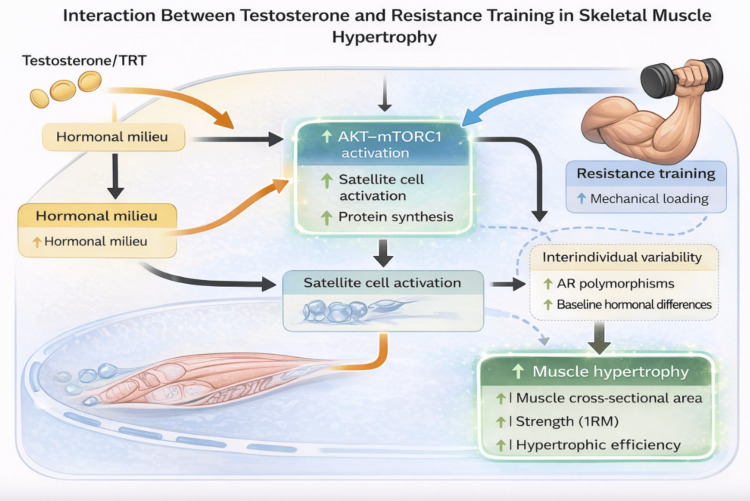
Interaction between testosterone and resistance training in skeletal muscle hypertrophy. Conceptual schematic illustrating the synergistic interaction between testosterone and resistance training in promoting skeletal muscle hypertrophy. Testosterone and testosterone replacement therapy (TRT) optimize the hormonal milieu, enhancing anabolic signaling pathways such as AKT–mTORC1 activation, increasing satellite cell activation, and sustaining muscle protein synthesis. Resistance training provides the primary mechanical stimulus through mechanical loading and mechanotransduction, which converges with androgen-mediated signaling to amplify hypertrophic responses. Interindividual variability is influenced by factors such as androgen receptor polymorphisms and baseline hormonal differences. Information was synthesized from references [7-14]. Image credits: The image was created by the authors using BioRender.

Collectively, this body of evidence establishes testosterone as a key modulator of resistance training-induced hypertrophy, capable of enhancing both the magnitude and efficiency of skeletal muscle adaptation (Figure 3). While supraphysiologic androgen exposure demonstrates the upper limits of anabolic potential, physiologically dosed testosterone primarily acts to optimize the biological environment in which resistance training-driven adaptations occur. As illustrated in Figure 3, these interactions provide a mechanistic and functional framework for understanding how testosterone and TRT may influence muscle hypertrophy and recovery, particularly in contexts where endogenous androgen signaling is impaired or insufficient to support optimal adaptation.

Effects of Testosterone on Tendon, Bone, and Musculoskeletal Integrity

Beyond its well-characterized anabolic actions in skeletal muscle, testosterone contributes to the structural integrity and recovery potential of tendons and bone, two tissues that critically determine load tolerance, injury risk, and long-term performance in physically active populations. From a mechanistic standpoint, androgen signaling appears to influence connective tissue remodeling by modulating extracellular matrix turnover, cellular proliferation, and the balance between synthesis and degradation of collagen-rich structures [12,13]. This is clinically relevant because tendon and enthesis injuries frequently arise from repetitive loading, impaired remodeling capacity, or incomplete restoration of mechanical properties following injury.

Experimental data suggest that androgens stimulate collagen synthesis and fibroblast activity, supporting extracellular matrix deposition and remodeling during tendon healing [12,13]. In this framework, adequate androgen signaling may promote a more efficient reparative response after tendon microdamage by enhancing the regenerative cellular response and improving matrix organization. However, the translational relevance of these effects requires careful interpretation: tendon healing quality is not determined solely by the amount of collagen produced but also by collagen alignment, cross-linking, and the restoration of tissue viscoelastic properties. Therefore, androgen-mediated effects should be conceptualized as potentially improving the biological substrate for repair, while the ultimate functional outcome remains dependent on mechanical loading conditions and rehabilitation protocols.

Testosterone also plays a central role in bone homeostasis through direct androgen receptor-mediated effects on osteoblast function and indirect effects via aromatization to estradiol, which contributes to suppression of bone resorption [14,15]. In clinical settings, TRT has been associated with improvements in bone mineral density, particularly at the lumbar spine and hip, alongside favorable changes in bone turnover markers, findings that support a net positive effect on skeletal integrity in populations with impaired androgen status [16]. For athletes and physically active individuals, these effects are clinically meaningful because bone strength and remodeling efficiency influence stress fracture susceptibility, recovery time after injury, and the ability to sustain high training loads without structural failure.

In the context of sports injury and orthopedic reconstruction, the tendon-bone interface represents a biologically complex and mechanically critical region. Healing at this enthesis involves coordinated collagen deposition, fibrocartilage formation, and gradual restoration of load transfer properties. Mechanistic frameworks propose that testosterone may support tendon-bone integration by promoting collagen synthesis and facilitating fibrocartilage development at the interface, potentially enhancing graft incorporation and biomechanical stability after reconstruction [17]. These concepts are particularly relevant for procedures such as ligament reconstruction, where delayed biological integration can prolong rehabilitation timelines and increase the risk of reinjury. Nonetheless, while mechanistic rationale is strong and preclinical signals are encouraging, robust athlete-specific clinical data remain limited, and the degree to which these biological effects translate into clinically meaningful reductions in reinjury or improvements in return-to-play outcomes remains uncertain [17].

Taken together, available evidence supports the view that testosterone exerts tissue-level effects extending beyond skeletal muscle, influencing connective tissue and skeletal remodeling processes that underpin overall musculoskeletal durability (Table 1). In rehabilitation contexts, particularly in individuals with reduced endogenous androgen signaling, TRT may contribute to a more favorable biological environment for tendon and bone recovery by supporting collagen turnover, osteoblastic activity, and tendon-bone integration. As summarized in Table 1, these effects are mechanistically plausible and clinically relevant; however, their functional impact is likely contingent on appropriate mechanical loading, rehabilitation design, and a clear distinction between physiological hormone replacement and supraphysiologic androgen exposure. Future athlete-focused studies are needed to determine whether optimization of androgen status meaningfully improves tendon healing quality, enhances bone structural outcomes, and, ultimately, reduces reinjury risk under real-world training demands.

**Table 1:** Effects of testosterone on tendon, bone, and musculoskeletal integrity. Summary of proposed biological effects of testosterone on tendon, bone, and overall musculoskeletal integrity, highlighting mechanistic pathways and their potential functional relevance in physically active populations. The table integrates experimental, mechanistic, and clinical evidence supporting the role of androgen signaling in connective tissue remodeling and skeletal health, while acknowledging that translation to athlete-specific clinical outcomes remains incompletely defined.

Tissue/Structure	Proposed biological effects of testosterone	Functional and clinical relevance	Key references
Tendon	Increased collagen synthesis, enhanced fibroblast activity, and modulation of extracellular matrix remodeling	Improved tendon healing capacity, enhanced load tolerance, and potential reduction in injury persistence	[12,13]
Bone	Direct androgen receptor–mediated stimulation of osteoblast activity and indirect suppression of bone resorption via aromatization to estradiol	Increased bone mineral density, improved skeletal integrity, and reduced susceptibility to stress fractures	[14-16]
Tendon–bone interface (enthesis)	Promotion of collagen deposition and fibrocartilage formation at the tendon–bone junction	Enhanced graft incorporation and biomechanical stability following orthopedic reconstruction	[17]
Connective tissue remodeling	Improved balance between anabolic and catabolic processes governing collagen turnover	Structural durability during rehabilitation and adaptation to repetitive mechanical loading	[12-15]
Musculoskeletal integrity (global)	Coordinated effects on muscle–tendon–bone unit supporting tissue regeneration and remodeling	Improved recovery potential and resilience to reinjury under training demands	[12-17]

Immunomodulatory and Anti-inflammatory Effects of Testosterone in Tissue Repair

Testosterone exerts clinically relevant immunomodulatory effects that influence the inflammatory milieu during tissue repair following sports-related injury. Inflammation represents a critical early phase of healing, coordinating debris clearance and activation of regenerative pathways; however, excessive intensity or prolonged persistence of inflammatory signaling can impair regeneration, promote fibrosis, and delay functional recovery. Within this context, testosterone appears to play a regulatory role by modulating both innate immune responses and downstream oxidative stress pathways [18,19].

At the cytokine level, testosterone suppresses key pro-inflammatory mediators, including tumor necrosis factor-α, interleukin (IL)-1β, and IL-6, while upregulating the anti-inflammatory cytokine IL-10 [18,19]. This shift toward an anti-inflammatory cytokine profile contributes to attenuation of excessive inflammatory signaling without abolishing the acute inflammatory response required for effective tissue repair. Such modulation is particularly relevant in musculoskeletal injury, where persistent low-grade inflammation has been implicated in impaired muscle regeneration, delayed tendon healing, and pathological scar formation.

Beyond cytokine regulation, testosterone influences immune cell phenotype and function, particularly macrophage polarization. Experimental evidence indicates that androgen signaling promotes a shift toward the reparative M2 macrophage phenotype, which is associated with tissue remodeling, extracellular matrix reorganization, and resolution of inflammation [20]. M2 macrophages secrete growth factors and anti-inflammatory mediators that support myofiber regeneration, fibroblast coordination, and angiogenesis, thereby facilitating the transition from inflammatory to regenerative phases of healing. This macrophage-mediated mechanism provides a cellular framework through which testosterone may indirectly enhance structural recovery across muscle and connective tissues.

Oxidative stress represents an additional biological axis through which inflammation can compromise tissue repair. Reactive oxygen species (ROS), while necessary at low levels for cellular signaling, can induce cellular damage, lipid peroxidation, and mitochondrial dysfunction when produced in excess. Testosterone has been shown to enhance endogenous antioxidant defenses by increasing the activity of enzymes such as superoxide dismutase and catalase, thereby mitigating ROS-induced damage during periods of injury, immobilization, or intense training stress [21]. By limiting oxidative injury, testosterone may help preserve cellular integrity and optimize the regenerative environment required for effective tissue remodeling.

**Figure 4:**
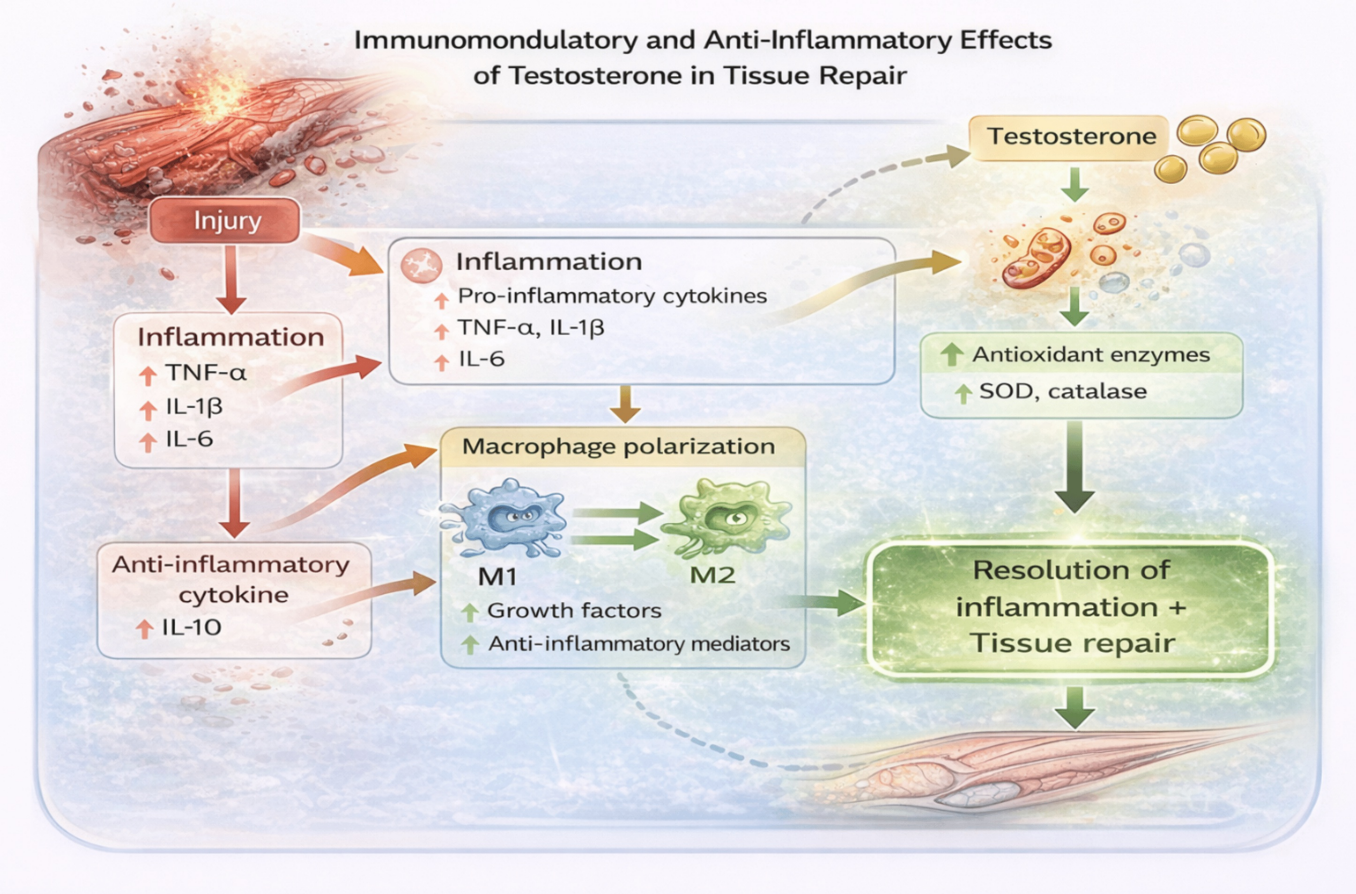
Immunomodulatory and anti-inflammatory effects of testosterone in tissue repair. Schematic illustration of the immunomodulatory mechanisms through which testosterone influences tissue repair following musculoskeletal injury. Testosterone attenuates excessive inflammation by suppressing pro-inflammatory cytokines (tumor necrosis factor-α (TNF-α), interleukin (IL)-1β, IL-6) while promoting anti-inflammatory signaling via IL-10. Concurrently, androgen signaling facilitates macrophage polarization toward the reparative M2 phenotype and enhances endogenous antioxidant defenses, including superoxide dismutase (SOD) and catalase, supporting resolution of inflammation and effective tissue regeneration. Information was synthesized from references [18-21]. Image credits: The image was created by the authors using BioRender.

Collectively, these immunomodulatory and antioxidant actions position testosterone as a regulator of inflammatory balance during tissue repair rather than as a purely anti-inflammatory agent (Figure 4). As illustrated in Figure 4, testosterone modulates the post-injury inflammatory milieu by suppressing excessive pro-inflammatory cytokine signaling, promoting macrophage polarization toward the reparative M2 phenotype, and enhancing endogenous antioxidant defenses. These coordinated effects may facilitate the transition from inflammation to regeneration, limit fibrotic remodeling, and support efficient tissue healing when androgen signaling is adequate. However, the clinical relevance of these mechanisms remains context dependent, influenced by injury severity, timing of intervention, and rehabilitation strategy, underscoring the need for careful integration of hormonal modulation within comprehensive recovery programs.

Evidence in Athletes and Physically Active Populations

Evidence evaluating the role of TRT in athletes and physically active populations remains limited but increasingly relevant, particularly outside elite competitive settings. In non-elite and recreational athletes, TRT has been explored as a therapeutic strategy to optimize musculoskeletal recovery, preserve bone integrity, and support sustained physical performance in the setting of clinically meaningful androgen deficiency [1,4]. By restoring physiological androgen levels, TRT may enhance muscle regeneration, support connective tissue remodeling, and facilitate rehabilitation following injury, rather than serving as a direct performance-enhancing intervention.

From a practical standpoint, currently available TRT formulations, including intramuscular injections, transdermal gels or patches, oral testosterone undecanoate, and long-acting subcutaneous or intramuscular preparations, are designed to approximate endogenous testosterone exposure and avoid supraphysiologic peaks [5,6,10]. When appropriately prescribed and monitored, these delivery systems aim to stabilize circulating androgen concentrations within the physiological range, thereby supporting muscle function, recovery capacity, and metabolic health without replicating the pharmacokinetic profiles associated with androgen misuse or abuse. This distinction is particularly important when interpreting outcomes related to musculoskeletal adaptation and injury recovery.

Regulatory context further differentiates therapeutic TRT from ergogenic androgen use. In professional and elite sport, testosterone and related compounds are prohibited under World Anti-Doping Agency (WADA) regulations unless a Therapeutic Use Exemption (TUE) is granted, reflecting concerns related to fairness rather than an absence of biological or clinical effect [2,22-24]. In contrast, recreational athletes and physically active individuals are not subject to anti-doping enforcement, allowing TRT to be considered within a conventional medical framework focused on symptom resolution, injury recovery, and long-term musculoskeletal health rather than competitive advantage [25,26].

Observational evidence from military, endurance, and high-training-load populations provides indirect but biologically coherent support for the relevance of androgen status in physically active individuals. Studies have demonstrated that low baseline testosterone levels are associated with increased incidence of stress fractures, impaired bone remodeling, delayed recovery from training stress, and higher susceptibility to overuse injuries [26]. These findings suggest that androgen deficiency may compromise the integrated muscle-tendon-bone unit, particularly under conditions of repetitive mechanical loading, caloric deficit, or prolonged physiological stress.

Within this framework, TRT may contribute to improved injury resilience by enhancing muscle-tendon-bone integrity, supporting collagen turnover, preserving bone density, and mitigating oxidative stress during rehabilitation and return-to-activity phases [3,15,27]. Importantly, these potential benefits are most plausibly realized in individuals with demonstrable androgen deficiency, rather than in eugonadal athletes, and should be interpreted as facilitative of recovery rather than as direct enhancers of athletic performance.

Collectively, available evidence supports a cautious but biologically plausible role for TRT in selected physically active populations, particularly where low testosterone contributes to impaired recovery, recurrent injury, or delayed rehabilitation. However, high-quality prospective studies specifically targeting athletic cohorts remain scarce, and current conclusions rely largely on extrapolation from mechanistic data, observational studies, and non-athlete clinical populations. As such, TRT in athletes should be framed as a targeted therapeutic intervention guided by endocrine evaluation, clinical symptoms, and functional impairment, rather than as a generalized strategy for performance optimization.

Clinical Implications for Injury Recovery and Rehabilitation

The translational rationale for testosterone and testosterone-based therapies in injury recovery centers on their capacity to influence multiple determinants of rehabilitation success: preservation of lean mass during catabolic stress, enhancement of regenerative capacity after tissue injury, optimization of neuromuscular function, and modulation of inflammatory and oxidative pathways that shape healing quality. Preclinical evidence supports these concepts, demonstrating that testosterone can accelerate myofiber regeneration, reduce fibrotic remodeling, and improve structural restoration of injured muscle tissue, biological effects that, if translated clinically, would be expected to shorten recovery timelines and improve functional outcomes after musculoskeletal injury [23].

Clinically, one of the most consistent and actionable implications relates to the prevention or attenuation of disuse atrophy during immobilization. Periods of reduced loading, whether due to injury, postoperative restrictions, or inactivity, are characterized by rapid net protein loss and reduced muscle strength, often out of proportion to the duration of immobilization. In this context, TRT has been associated with improved muscle protein synthesis and preservation of muscle function, providing a mechanistic basis for its potential use as an adjunct in rehabilitation paradigms where endogenous androgen signaling is impaired. Ferrando et al. reported improved muscle strength and protein synthesis in older hypogonadal men receiving testosterone during inactivity, underscoring the relevance of androgen status in mitigating the catabolic effects of disuse and facilitating return of function [9]. Importantly, these findings highlight the potential role of TRT not as a substitute for rehabilitation but as a biological support that preserves the substrate upon which rehabilitation can act.

Beyond testosterone itself, the clinical signal supporting anabolic support during recovery is reinforced by evidence involving anabolic derivatives. Oxandrolone, for example, has been shown to preserve lean mass and accelerate rehabilitation in trauma and burn populations, settings characterized by profound hypercatabolism, systemic inflammation, and high risk of prolonged functional decline [24]. While these data are indirect and derived from non-athletic cohorts, they provide proof-of-concept that androgen-based interventions can meaningfully affect recovery trajectories when catabolic pressure is high. Taken together, these observations support the rationale that selected patients experiencing catabolic stress, prolonged immobilization, or impaired anabolic signaling may benefit from carefully supervised anabolic support as part of a multidisciplinary rehabilitation strategy.

Testosterone’s potential relevance to rehabilitation also extends beyond muscle size to neuromuscular performance. Recovery is ultimately determined by the restoration of force production, motor coordination, and movement efficiency, outcomes influenced by neural drive, motor unit recruitment, and muscle contractile capacity. Testosterone may improve neuromuscular coordination during rehabilitation by enhancing motor neuron excitability and muscle force generation, providing a plausible mechanism through which strength and functional performance improvements may exceed what would be expected from hypertrophy alone [25]. This is particularly relevant in later rehabilitation stages, where functional performance and movement quality, not only muscle mass, determine return-to-activity readiness.

In addition, testosterone’s immunomodulatory and antioxidant effects may reduce secondary injury cascades that impair recovery quality. Excessive or prolonged inflammation can promote fibrosis and inhibit optimal regeneration, while oxidative stress contributes to cellular damage and delayed tissue remodeling. By attenuating inflammatory signaling and strengthening antioxidant defenses, testosterone may support a more favorable healing environment and reduce maladaptive remodeling processes that prolong pain, stiffness, or functional limitation [19]. These effects may be especially relevant in injury contexts characterized by high inflammatory burden or repeated microtrauma during early return-to-load phases.

In orthopedic and perioperative contexts, the clinical implications are conceptually similar. Postoperative recovery often involves immobilization, reduced loading, systemic inflammatory stress, and negative nitrogen balance, conditions that favor muscle loss and delayed strength restoration. TRT has been associated with improved strength recovery and reduced atrophy after periods of reduced activity, suggesting potential value in selected patients undergoing surgery when androgen deficiency is present and clinically relevant [9,25]. Moreover, anabolic therapies have been linked to improved perioperative nitrogen balance and reduced inflammatory catabolic burden, potentially enhancing postoperative recovery and rehabilitation progression in high-risk settings [24]. While athlete-specific perioperative evidence remains limited, these mechanisms provide a coherent framework for considering endocrine optimization as part of comprehensive recovery planning.

Finally, sustained androgen signaling may contribute to longer-term injury resilience by preserving lean mass, supporting tendon quality, and maintaining bone density, factors closely associated with mechanical durability and reduced reinjury risk in physically active individuals [1,14]. In this framework, the primary rehabilitative value of TRT is not simply accelerating short-term recovery but reducing vulnerability during the transition back to training by maintaining structural integrity across the muscle-tendon-bone unit.

Collectively, current evidence supports a biologically plausible and clinically relevant role for testosterone and TRT as adjunctive strategies in recovery and rehabilitation, particularly in individuals with demonstrable androgen deficiency or high catabolic burden. The potential clinical contexts, mechanisms, and limitations of these interventions are summarized in Table 2, which highlights scenarios in which androgen optimization may facilitate rehabilitation outcomes when appropriately integrated into multidisciplinary care. However, these interventions should be framed as targeted, medically supervised supports within a rehabilitation program, rather than as universal tools for performance enhancement. Future studies focused on athletic cohorts, clinically meaningful functional outcomes, and safety monitoring are needed to define which subgroups derive the greatest benefit and to establish best practices for integrating TRT into sports medicine rehabilitation pathways.

**Table 2:** Potential clinical implications of testosterone and testosterone replacement therapy in injury recovery and rehabilitation. Summary of proposed clinical contexts in which testosterone and testosterone replacement therapy (TRT) may influence injury recovery and rehabilitation outcomes. The table integrates mechanistic, clinical, and observational evidence supporting potential benefits while highlighting key considerations and limitations. These applications should be interpreted as adjunctive and context-dependent, rather than universal strategies for performance enhancement.

Clinical context	Proposed role of testosterone/TRT	Potential benefit	Key considerations	Key references
Immobilization/Disuse	Attenuation of catabolic muscle loss and preservation of anabolic signaling during inactivity	Preservation of lean mass and muscle strength, facilitating rehabilitation progression	Applicable primarily in androgen-deficient individuals; TRT should complement, not replace, rehabilitation	[9,23]
Postoperative recovery	Support of anabolic balance and reduction of postoperative muscle atrophy	Improved strength recovery and reduced loss of muscle mass following surgery	Careful patient selection and monitoring required; evidence in athletes is indirect	[9,24,25]
Severe injury/trauma	Reduction of hypercatabolic response and support of protein synthesis	Accelerated rehabilitation and improved functional recovery trajectories	Evidence largely extrapolated from trauma and burn populations	[24]
Neuromuscular rehabilitation	Enhancement of motor neuron excitability and muscle force generation	Improved functional performance and coordination during later rehabilitation phases	Adjunctive role; functional gains depend on structured rehabilitation programs	[25]
Long-term injury resilience	Maintenance of muscle–tendon–bone integrity and anabolic milieu	Reduced reinjury risk and improved musculoskeletal durability	Physiological dosing essential; long-term outcomes in athletes remain under investigation	[1,14]

Ergogenic and Systemic Performance Effects of Testosterone

Beyond its localized effects on skeletal muscle and connective tissues, testosterone exerts systemic physiological actions that influence performance capacity, recovery efficiency, and metabolic resilience. One of the most consistently documented systemic effects of testosterone is its role in erythropoiesis. Testosterone stimulates red blood cell production through increased erythropoietin signaling and modulation of iron metabolism, leading to elevations in hemoglobin concentration and hematocrit [28]. These hematological adaptations enhance oxygen-carrying capacity and may improve aerobic work capacity, particularly in individuals with low baseline testosterone or impaired erythropoietic function. In recovery contexts, improved oxygen delivery may also support tissue repair processes and mitigate fatigue during rehabilitation and return-to-training phases.

Testosterone additionally influences metabolic regulation and body composition, factors that indirectly but meaningfully affect athletic performance. Clinical and observational studies indicate that testosterone reduces visceral adiposity and improves insulin sensitivity, contributing to a more favorable metabolic profile [29,30]. These changes can optimize power-to-weight ratio, reduce cardiometabolic strain during training and recovery, and improve energy utilization under physical stress. Importantly, such metabolic effects are most evident in hypogonadal or metabolically compromised populations, underscoring the context-dependent nature of testosterone’s systemic benefits.

At the cellular level, testosterone has been associated with enhanced mitochondrial biogenesis and increased activity of oxidative enzymes within skeletal muscle, mechanisms that may favor endurance adaptations and improve fatigue resistance [31-34]. These effects provide biological plausibility for improved aerobic performance; however, translation to measurable endurance gains in athletic populations remains inconsistent. Most supportive data derive from aging or hypogonadal cohorts, where restoration of physiological androgen levels corrects underlying deficits rather than conferring supraphysiologic advantage [1,5]. Consequently, while testosterone may normalize endurance capacity in deficient individuals, its ability to enhance endurance beyond baseline physiological limits appears limited and highly variable.

The ergogenic effects of testosterone are most robust and reproducible in domains requiring maximal force production, rapid power generation, and anaerobic performance. Controlled trials in healthy men demonstrate significant improvements in maximal strength, 1RM, sprint performance, vertical jump height, and anaerobic capacity following testosterone or anabolic steroid administration [35-37]. These performance gains arise from a combination of muscle hypertrophy, increased muscle cross-sectional area, and neural adaptations, including enhanced motor unit recruitment and firing rates. Such neural contributions help explain why strength and power improvements may precede or exceed gains predicted by muscle mass alone.

In contrast, evidence for testosterone-mediated enhancement of endurance performance remains mixed. Although increases in hemoglobin and oxygen transport theoretically support aerobic capacity, observed improvements in maximal oxygen uptake (VO₂max) are inconsistent and appear highly dependent on baseline hormonal status, training level, and study design [38]. In well-trained or eugonadal individuals, endurance performance may be constrained more by cardiovascular, pulmonary, or metabolic factors than by androgen availability, limiting the ergogenic impact of testosterone in this domain. Accordingly, testosterone’s performance-enhancing profile is best characterized as discipline-specific, with clear benefits in strength- and power-oriented activities and more modest or variable effects on endurance performance [39].

Taken together, testosterone exerts broad systemic effects that intersect with performance through hematological, metabolic, mitochondrial, muscular, and neural pathways. However, the magnitude and clinical relevance of these effects are strongly context dependent, influenced by baseline androgen status, training characteristics, and physiological reserve. From a sports medicine perspective, these findings reinforce the importance of distinguishing therapeutic normalization of testosterone levels from supraphysiologic androgen exposure, as the latter disproportionately amplifies ergogenic outcomes while introducing substantial ethical, regulatory, and safety concerns. In rehabilitation and recovery settings, the systemic actions of testosterone may therefore be most appropriately viewed as facilitators of physiological normalization and resilience rather than as primary drivers of performance enhancement.

Ethical, Regulatory, and Clinical Considerations in Athletes

The use of testosterone in athletic populations requires careful ethical, regulatory, and clinical framing, given the historical association of androgens with performance enhancement and misuse. Nevertheless, accumulating mechanistic, clinical, and observational evidence indicates that TRT, when appropriately indicated and medically supervised, may play a legitimate therapeutic role in selected athletic populations. Central to this discussion is the need to clearly distinguish evidence-based TRT aimed at restoring physiological androgen levels from non-therapeutic androgen use intended to confer competitive advantage.

Contemporary consensus statements in sports endocrinology acknowledge that athletes exposed to high training volumes, relative energy deficiency, prolonged caloric restriction, or sustained physiological stress may develop functional suppression of the hypothalamic-pituitary-gonadal axis [40]. This adaptive or maladaptive hormonal suppression has been associated with impaired recovery capacity, reduced bone mineral density, altered body composition, and increased susceptibility to stress fractures and overuse injuries. In such cases, correction of underlying contributors, such as energy availability, training load, sleep, and psychological stress, remains the first-line intervention. However, consensus frameworks recognize that in carefully selected individuals, persistent androgen deficiency may continue to contribute to clinically meaningful dysfunction despite optimization of modifiable factors, raising the question of whether targeted hormonal replacement may be appropriate [40].

From a regulatory perspective, testosterone and related compounds remain prohibited in competitive athletes under WADA regulations unless a TUE is granted [41]. This regulatory stance reflects principles of fairness and integrity in sport rather than a categorical rejection of testosterone’s therapeutic potential. The TUE process underscores the importance of rigorous endocrine evaluation, documentation of medical necessity, and ongoing monitoring to ensure that TRT is prescribed solely to restore physiological function and not to enhance performance beyond normal limits. Consequently, regulatory compliance should be viewed as a framework for responsible clinical decision-making rather than as a barrier to legitimate medical care.

A further ethical consideration involves the distinction between medically supervised TRT and androgen misuse or abuse. Recent reviews emphasize that many of the adverse outcomes historically attributed to testosterone, including erythrocytosis, cardiovascular risk, infertility, dyslipidemia, and long-term suppression of endogenous testosterone, are predominantly associated with supraphysiologic dosing, polypharmacy, or non-medical use [42]. In contrast, TRT prescribed within physiological ranges, guided by established clinical indications and accompanied by appropriate monitoring, demonstrates a substantially different risk profile. This distinction is critical to avoid conflating therapeutic hormone replacement with practices that fall outside accepted medical and ethical standards.

Clinically, the potential role of TRT in athletes must therefore be framed as adjunctive and individualized. In non-elite athletes and physically active individuals not subject to anti-doping enforcement, TRT may represent a supportive intervention aimed at restoring hormonal homeostasis, facilitating recovery, preserving musculoskeletal health, and improving quality of life when androgen deficiency is clearly documented. In competitive athletes, any consideration of TRT requires strict adherence to regulatory oversight, transparent documentation, and multidisciplinary collaboration involving sports physicians, endocrinologists, and regulatory bodies [40-42].

Ultimately, ethical use of TRT in athletic contexts depends on proportionality, transparency, and clinical justification. TRT should not be viewed as a tool for performance enhancement, but rather as a targeted medical therapy reserved for cases in which androgen deficiency contributes to impaired health, delayed recovery, or increased injury risk. Future research focusing on athlete-specific outcomes, long-term safety, and ethical implementation frameworks will be essential to refine best practices and ensure that TRT, when used, aligns with both medical ethics and the integrity of sport.

Discussion

This narrative review integrates molecular, physiological, clinical, and ethical evidence to examine the role of testosterone and TRT in musculoskeletal recovery, rehabilitation, and performance contexts relevant to athletes and physically active populations [7-9,12,18,40]. Across multiple biological domains, testosterone appears to function as an important regulatory hormone influencing skeletal muscle hypertrophy, connective tissue remodeling, bone integrity, inflammatory balance, and systemic metabolic capacity [7,8,12-14,18,28]. Rather than acting as an isolated driver of performance enhancement, the cumulative evidence supports a more nuanced interpretation in which testosterone primarily serves a permissive and modulatory role, optimizing the biological environment required for effective adaptation and recovery [7,8,10,39,42].

At the molecular and cellular level, testosterone exerts coordinated genomic and non-genomic actions that promote muscle protein synthesis, satellite cell activation, and anabolic signaling through pathways such as AKT-mTORC1, while concurrently suppressing catabolic regulators including myostatin and ubiquitin-proteasome activity [7-11]. These mechanisms provide a biologically coherent explanation for the dose-response relationships observed between androgen exposure and skeletal muscle hypertrophy, particularly when testosterone signaling converges with mechanical loading during resistance training [7,8,9,10,12]. However, much of this mechanistic and experimental evidence derives from controlled studies in non-athletic, hypogonadal, or aging populations, limiting direct extrapolation to competitive or well-trained athletes with preserved endocrine function [8-10,13,14].

Beyond skeletal muscle, testosterone’s influence on tendon, bone, and the tendon-bone interface highlights its broader relevance to musculoskeletal durability and injury resilience [12-15,17]. Experimental and clinical data suggest that adequate androgen signaling supports collagen synthesis, osteoblastic activity, and extracellular matrix remodeling, processes fundamental to tissue healing after injury or surgical reconstruction [12-17]. In parallel, testosterone’s immunomodulatory and antioxidant effects may contribute to the regulation of the inflammatory milieu during tissue repair, facilitating the resolution of inflammation and limiting maladaptive fibrotic remodeling [18-21]. Nevertheless, translation of these tissue-level effects into clinically meaningful outcomes in athlete-specific cohorts remains incompletely defined and likely depends on appropriate mechanical loading, rehabilitation design, and timing of intervention [17,26].

Systemically, testosterone influences erythropoiesis, metabolic regulation, mitochondrial function, and neural activation, intersecting with multiple determinants of performance and recovery capacity [28-32]. The ergogenic effects of testosterone are most consistently demonstrated in strength- and power-based outcomes, including maximal force production and anaerobic performance [35-37]. In contrast, endurance-related benefits are more variable, with inconsistent effects on VO₂max and aerobic performance in eugonadal or well-trained individuals [38,39]. These findings underscore the importance of distinguishing therapeutic normalization of androgen levels from supraphysiologic androgen exposure, the latter disproportionately amplifying ergogenic effects while introducing substantially greater ethical, regulatory, and safety concerns [39,41,42].

From an ethical and clinical perspective, the use of TRT in athletic contexts requires careful individualization, rigorous endocrine evaluation, and clear separation from non-therapeutic androgen use [40-42]. Consensus statements acknowledge that athletes exposed to high training loads or relative energy deficiency may develop functional suppression of the hypothalamic-pituitary-gonadal axis, which has been associated with impaired recovery, reduced bone density, and increased injury risk [40]. In selected cases, TRT may represent a legitimate adjunctive therapy when prescribed within physiological ranges and integrated into a multidisciplinary rehabilitation framework [1,4-6,40]. However, regulatory oversight, particularly in competitive athletes subject to anti-doping regulations, remains essential to safeguard both athlete health and the integrity of sport [41].

Importantly, high-quality prospective trials specifically conducted in injured athletic cohorts remain scarce. Much of the available clinical and translational evidence is derived from hypogonadal, aging, military, or high-catabolic-burden populations, necessitating cautious extrapolation when interpreting applicability to competitive athletes. Additionally, sex-specific applicability represents a key limitation, as the available clinical TRT evidence base is predominantly male, whereas data in female athletes remain limited and cannot be directly extrapolated from male cohorts. This evidence gap underscores the need for athlete-centered research designs incorporating standardized functional outcomes, return-to-play metrics, and long-term safety monitoring.

**Figure 5:**
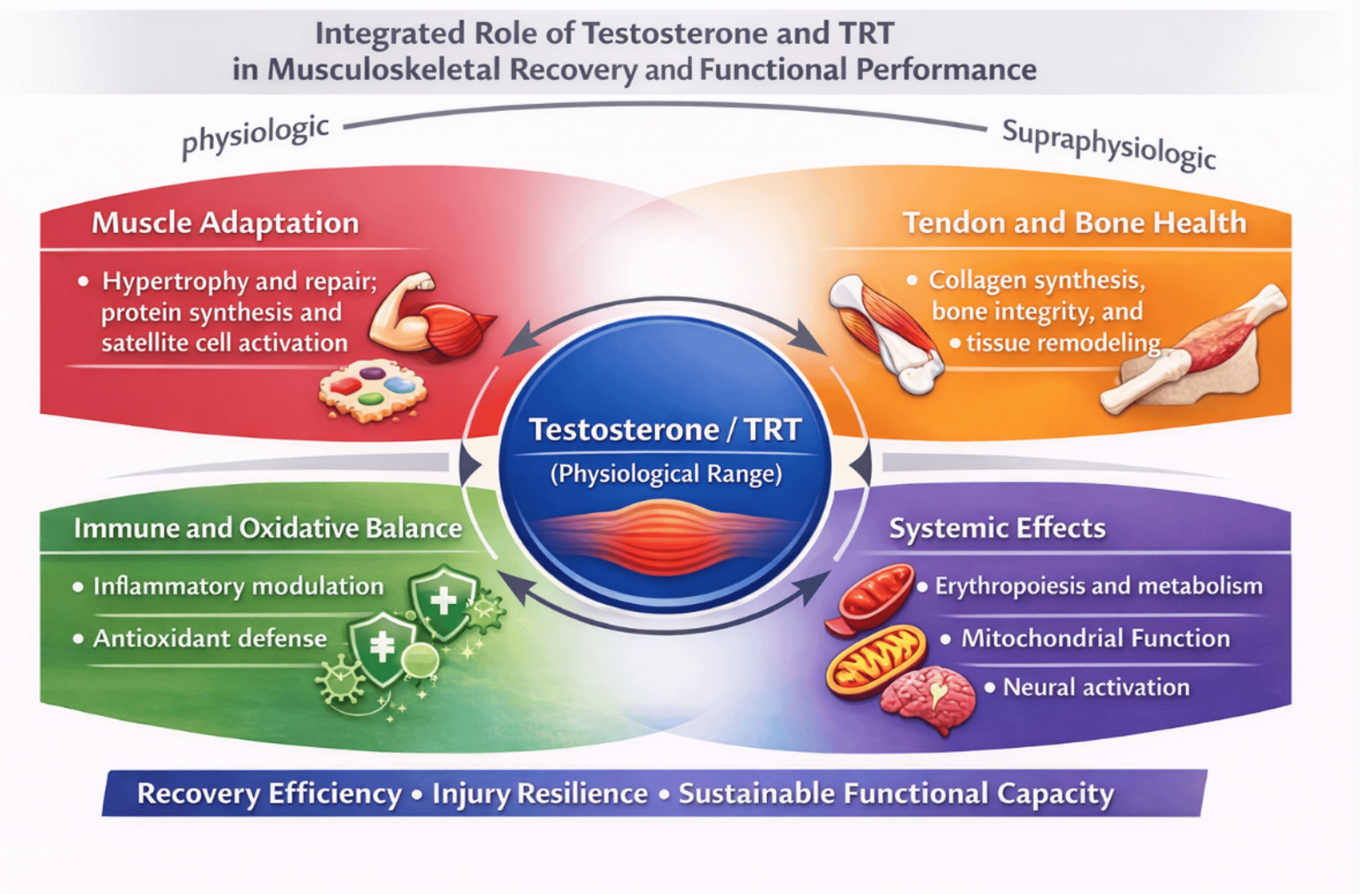
Integrated role of testosterone and testosterone replacement therapy in musculoskeletal recovery and performance. Conceptual schematic illustrating the integrated, multi-system role of testosterone and testosterone replacement therapy (TRT) within physiological ranges in musculoskeletal recovery, rehabilitation, and functional performance. Testosterone acts as a central regulatory hormone influencing skeletal muscle adaptation through enhancement of protein synthesis, satellite cell activation, and hypertrophic remodeling; tendon and bone health via support of collagen synthesis and extracellular matrix remodeling; immune and oxidative balance through modulation of inflammatory signaling and reinforcement of antioxidant defenses; and systemic effects including erythropoiesis, metabolic regulation, mitochondrial function, and neural activation. Collectively, these coordinated actions contribute to improved recovery efficiency, injury resilience, and sustainable functional capacity, emphasizing testosterone’s permissive and modulatory role rather than its function as an isolated driver of performance enhancement. Information was synthesized from references [7-21]. Image credits: The image was created by the authors using BioRender.

Overall, certainty of evidence varies across biological domains, being highest for anabolic and strength-related outcomes in controlled settings and lowest for athlete-specific functional endpoints, where data remain indirect and heterogeneous [39,40,42]. Future research should prioritize athlete-focused cohorts, clinically meaningful functional outcomes, long-term safety monitoring, and ethically grounded implementation frameworks to more precisely define the role of testosterone and TRT within sports medicine and rehabilitation practice [40,42] (Figure 5).

## Conclusions

This narrative review highlights testosterone as a permissive, systems-level regulator of musculoskeletal recovery rather than an isolated driver of performance enhancement. Across molecular, tissue, and systemic domains, adequate androgen signaling appears to support skeletal muscle remodeling, connective tissue integrity, inflammatory balance, and overall recovery capacity, providing a coherent biological framework for its relevance in rehabilitation contexts. However, translation of these mechanisms to athlete-specific outcomes remains limited by heterogeneous evidence and a scarcity of prospective data in well-trained populations. Accordingly, TRT should be considered a targeted, medically supervised intervention reserved for individuals with documented androgen deficiency and clinically meaningful impairment, where restoration of physiological levels may facilitate recovery when integrated into structured rehabilitation. Future research should also address sex-specific outcomes, as current therapeutic TRT evidence remains predominantly derived from male populations, and data in female athletes are comparatively limited.
